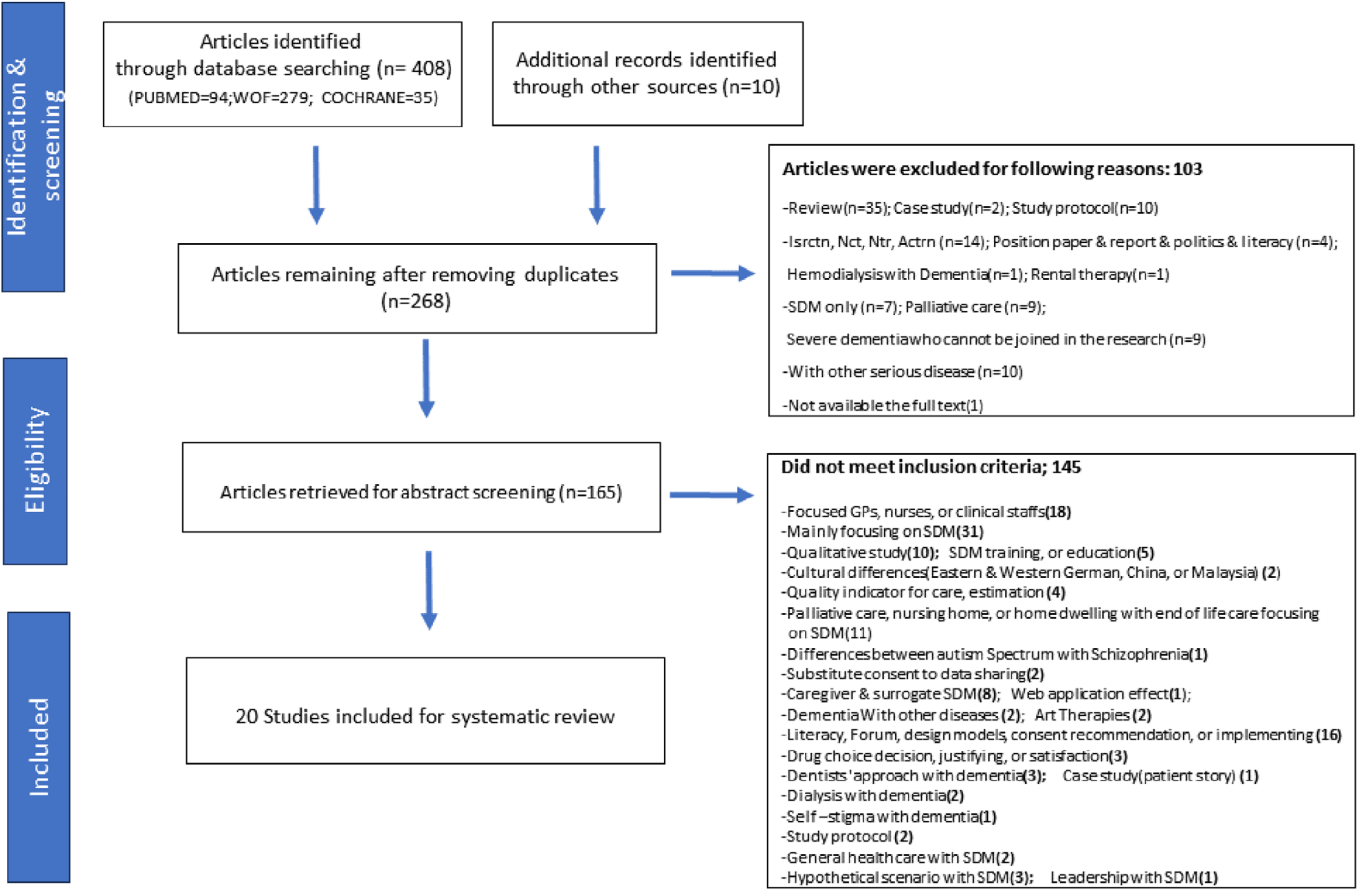# Preference Assessment in Dementia Care: Systematic Literature Reivew

**DOI:** 10.1002/alz.088108

**Published:** 2025-01-09

**Authors:** Hyejeen Lim, Young Min Choe, Jaeuk Hwang, Soo‐Hee Choi, Seong Jae Kim, Jung Hie Lee, Jong Inn Woo, Eun Hyun Seo

**Affiliations:** ^1^ Premedical Science, College of Medicine, Chosun University, Gwangju Korea, Republic of (South); ^2^ College of Education, Seoul National University, Seoul Korea, Republic of (South); ^3^ Hallym University Dongtan Sacred Heart Hospital, Hwaseong Korea, Republic of (South); ^4^ College of Medicine, Soonchunhyang University, Asan Korea, Republic of (South); ^5^ Associate Professor, Seoul National University College of Medicine, Seoul Korea, Republic of (South); ^6^ Chosun University Hospital, College of Medicine, Gwangju Korea, Republic of (South); ^7^ Professor Emeritus, Kangwon National University, College of Medicine, Chuncheon Korea, Republic of (South); ^8^ Professor Emeritus, Seoul National University College of Medicine, Seoul Korea, Republic of (South)

## Abstract

**Background:**

Boosting shared decision‐making (SDM) for individuals with dementia is a critical aspect of patient‐centered care. This collaborative approach respects the autonomy and dignity of patients, even when they face challenges in communication or decision‐making due to their medical condition. Understanding patients’ values and preferences may be central to a successful SDM process. The aim of this study is to review various preference evaluation methods used for individuals with dementia in clinical settings, and to suggest the most effective and feasible tools for accurately measuring values and preferences.

**Methods:**

We strictly followed an updated guideline for the Preferred Reporting Items for Systematic Reviews and Meta‐Analysis (PRISMA) (Page et al., 2021). A systematic review was conducted through a search of three online databases (PubMed, Web of Science, Cochrane) and included studies about patient preferences published in English between 2000 and 2023. This review included studies that used a quantitative approach including surveys and structured interviews to gather data on the preferences of individuals with dementia and cognitive impairment. Specific inclusion and exclusion criteria are shown in Figure 1.

**Results:**

After identification of 268 studies, twenty studies were finally eligible for review (Figure 1). Five categories of preference evaluations were generated from the review: (a) values and preferences in decision‐making (n = 6), (b) treatment goals (n = 2), (c) discrete choice experiments for preferences (n = 3), (d) diagnosis disclosure and communication (n = 5), (e) advanced care planning (n = 4). Most of the studies demonstrated substantial reliability and validity in their assessment methods for individuals with dementia.

**Conclusions:**

Our findings suggested that individuals with dementia can reliably express their values and preferences about treatment that they currently receive or will need in the future. Moreover, our review highlighted the need for flexible and adaptable assessment to accommodate each patient’s unique circumstances. The utilization of tools with the autonomy preference index, clearly defined treatment goals, and transparent diagnosis disclosure, seem to be critical for fostering effective SDM process.